# 

**Published:** 1956-04

**Authors:** 


MEDICAL JOURNAL OF THE SOUTH-WEST

*(Incorporating the Bristol Medico-Chirurgical Journal)*

Established 1883

VOL. 71 (ii) | April, 1956 | NO. 260

### Contents

*PUBLISHED QUARTERLY*

*ANNUAL SUBSCRIPTION* **16/-** *POST FREE*

PUBLISHED UNDER THE AUSPICES OF THE
BRISTOL MEDICO-CHIRURGICAL SOCIETY

### *Editorial Committee*

Dr. J. E. Cates, Department of Medicine, Bristol Royal Infirmary. *Editor.*
Dr. A. H. Gale, The University, Bristol 8. *Assistant Editor.*
Dr. J. Macrae, Ham Green Hospital. *Treasurer.*
Dr. N. J. Brown, Southmead Hospital, Bristol.
Mr. J. P. Partridge, Southmead Hospital, Bristol.

### *Local Correspondents*

*Bath* . . Mr. A. D. Bateman, 3 The Circus, Bath.
*Exeter* . . Dr. L. N. Jackson, Mount Jocelyn, Crediton, Devon.
*Gloucester* . Dr. R. F. Jarrett, 9 College Green, Gloucester.
*Plymouth* . Dr. C. R. Croft, Lexden, Hartley Av., Plymouth.
*Taunton* . Dr. M. McAlley, 1 The Crescent, Taunton.
*Torquay* . Dr. A. Robinson Thomas, 20 Devon Square, Newton Abbot, Devon.
*Truro* . . Dr. F. D. M. Hocking, St. Andrews, Perranporth, Cornwall.
*Yeovil* . . Mr. J. A. Vere Nicoll, Knapp House, Yeovil, Somerset.

### NOTICE TO CONTRIBUTORS

Original articles are invited, provided they have not been published elsewhere. Notes of forthcoming events, meetings held, degrees conferred, staff appointments, and other local news are welcomed. The editorial committee reserves the right to make literary corrections.

Illustrations should always be supplied ready for reproduction. All re-touchings or other operations required will be charged to the author. Line drawings should be drawn in Indian ink. We regret that we must ask authors to pay for making blocks, if this is necessary.

J. W. ARROWSMITH LTD., WINTERSTOKE ROAD,
BRISTOL.

### *Notes and News*

Dr. Ronald Woolmer, Reader in Anaesthetics, has been appointed Director of the Research Department in Anaesthetics of the Royal College of Surgeons of England. He was Chief Instructor at the European Centre (W.H.O.) for Anaesthesia at Copenhagen in 1951–52. For the past two years he has been Vice-Dean of the Faculty of Anaesthetists of the Royal College of Surgeons.

Dr. R. C. Wofinden has been appointed Medical Officer of Health for the City and County of Bristol in succession to Dr. R. H. Parry. He has been Deputy Medical Officer of Health since 1947. Dr. Wofinden qualified in 1937. After house posts at St. Mary's and at Rotherham he became successively Deputy Medical Officer of Health of Rotherham and of Bradford. In 1946 he won the Joseph Rogers Essay Prize, his essay being published in 1947 with the title, " Health Services in England ". Among his other publications is " Problem Families in Bristol " published in 1950.

Last year Professor Bruce Perry, at the invitation of the Rockefeller Foundation, spent three months in the United States to study methods of medical education.

Professor G. G. Lennon has been visiting professor at the University of Baghdad from January to April, 1956. On his way home he will be visiting Istanbul and Ankara to give lectures under the auspices of the British Council.

### UNIVERSITY OF BRISTOL

#### *PRIZES*

*Crosby Leonard Prize:* G. W. Manley.
*Francis Dudley Memorial Prize:* G. F. Lipscomb.
*Dental Gold Medal:* K. V. Mortimer.
*Mackie Pathology Prize:* W. E. Benney.

*M.D.* (*Bristol*)*:* Suzanne K. R. Clarke.

#### *M.B., CH.B.* (*BRISTOL*) (*DECEMBER*, 1955)

| | |
|---|---|
| A. B. Claudius-Cole. | F. J. M. Killick. |
| M. R. Clift. | Miss S. A. F. Maxted. |
| Miss I. R. Coulton. | Mrs. M. E. C. Parke. |
| A. Ganendran. | R. P. Saundby. |
| J. A. Hayes. | D. W. Seldon. |
| W. G. R. Hobbs. | J. A. C. Strachan. |
| D. I. D. Hodge. | M. T. White. |
| I. M. Joiner. | J. H. Williams. |

### Appointments

### UNITED BRISTOL HOSPITALS

| Name | Appointment | Formerly |
|---|---|---|
| MONTGOMERY, H., M.B., B.S. (LOND.). | Registrar in Pathology. | Resident Clinical Pathologist, St. Mary's Hospital, London. |
| MORGAN, E. J. R., B.D.S. (BRISTOL), M.B., CH.B. (BRISTOL). | Registrar in Dental Surgery. | Senior House Officer in Anaesthetics, United Bristol Hospitals. |

### SOUTH-WESTERN REGIONAL HOSPITAL BOARD

### BRISTOL

| Name | Appointment | Formerly |
|---|---|---|
| WRIGHT, D. W., M.B., CH.B. (BRISTOL). | Medical Registrar, Southmead Hospital. | S.H.O. in Medicine, Southmead Hospital. |
| TOVEY, J. E., M.B., CH.B. (BRISTOL). | Registrar in Pathology, Southmead Hospital. | S.H.O. in Clinical Pathology, Southmead Hospital. |
| MARTLEW, R. H., M.B., CH.B. (LIVERPOOL), D.P.M. (CONJOINT), M.D. (LIVERPOOL). | Consultant Psychiatrist, Bristol Mental Hospital, Fishponds, Bristol. | Senior Registrar, Rainhill Hospital, Liverpool. |
| WARNER, J., M.B., CH.B. (B'HAM). | Registrar in Psychiatry, Bristol Mental Hospitals. | S.H.O., Bristol Mental Hospitals. |

### BATH

| Name | Appointment | Formerly |
|---|---|---|
| SELIM, M., M.B., B.CH. (CAIRO), D.L.O. (CAIRO). | E.N.T. Registrar, Bath Group of Hospitals. | S.H.O. in E.N.T. Surgery, Barnet General Hospital. |
| CIANTAR, JOAN, M.B., B.CH. (WALES). | Registrar in Pathology, Bath Group of Hospitals. | Registrar in Pathology, Southend-on-Sea Group of Hospitals. |

### SOUTH SOMERSET

| Name | Appointment | Formerly |
|---|---|---|
| RUSSELL, HELEN M., M.R.C.S., L.R.C.P., M.A. (CANTAB), M.B., B.CHIR. (CANTAB.), D.OBST.R.C.O.G., M.R.C.O.G. | Clinical Assistant in Obstetrics and Gynaecology, South Somerset Clinical Area (8 sessions). | Senior Registrar in Obstetrics and Gynaecology, South Somerset Clinical Area. |

### DEVON AND CORNWALL

| Name | Appointment | Formerly |
|---|---|---|
| BUTLER, N. G. P., M.B., B.S., M.R.C.S., L.R.C.P., D.A., F.F.A.R.C.S. | Consultant Anaesthetist, Exeter Clinical Area. | Senior Anaesthetic Registrar, Portsmouth Group of Hospitals. |
| WEYHAUSEN, E. G., L.A.H. (DUBLIN), M.B., B.CH. (DUBLIN). | E.N.T. Registrar, South Devon and East Cornwall Hospital, Plymouth. | S.H.O. in E.N.T. Surgery, Clayponds Hospital, South Ealing. |
| REINOLD, D. G., M.R.C.S., L.R.C.P., PRIMARY F.R.C.S. | Surgical Registrar, Royal Cornwall Infirmary, Truro. | Locum Surgical Registrar at Royal Cornwall Infirmary. |
| WALKER, D. G., M.B., B.S. (SYDNEY), PRIMARY F.R.C.S. | Surgical Registrar, South Devon and East Cornwall Hospital, Plymouth. | Undertaking Final Fellowship. |

| *Name* | *Appointment* | *Formerly* |
|---|---|---|
| GUNATILLEKE, D. E., M.B., B.S. (CEYLON), F.R.C.S. (ENG.). | Registrar in Obstetrics and Gynaecology, Royal Devon and Exeter Hospital, Exeter. | Postgraduate work. |
| MUDREWICZ, J., M.B., CH.B. (EDIN.). | Registrar in Obstetrics and Gynaecology, Camborne–Redruth Miners' and General Hospital. | S.H.O. in Obstetrics, St. Mary's Maternity Hospital, West Croydon. |
| MANTLE, J. A., M.B., CH.B. (B'HAM), M.R.C.S., L.R.C.P., F.R.C.S. (ENG.) AND (EDIN.). | Consultant Orthopaedic Surgeon, West Cornwall Clinical Area. | Senior Registrar, Royal Orthopaedic Hospital, Birmingham. |
| PIGGOT, J., M.B., B.CH., B.A.O., F.R.C.S. (EDIN.). | Orthopaedic Registrar, Princess Elizabeth Orthopaedic Hospital, Exeter. | Registrar in General Surgery, Banbridge Hospital, Co. Down. |
| NERI, L. E., M.B., B.S. (MADRAS), F.R.C.S. (ENG.). | Senior Surgical Registrar to the Thoracic Unit at Hawkmoor Chest Hospital, Bovey Tracey. | Registrar in General Surgery, Whipps Cross Hospital. |

